# Pangenome-guided sequence assembly via binary optimization

**DOI:** 10.1093/bib/bbag084

**Published:** 2026-02-26

**Authors:** Josh Cudby, James Bonfield, Chenxi Zhou, Richard Durbin, Sergii Strelchuk

**Affiliations:** Department of Applied Mathematics and Theoretical Physics, University of Cambridge, Wilberforce Road, Cambridge CB3 0WA, United Kingdom; Department of Computer Science, University of Oxford, Parks Road, Oxford OX1 3QG, United Kingdom; Wellcome Sanger Institute, Wellcome Genome Campus, Hinxton, Cambridge CB10 1RQ, United Kingdom; Department of Genetics, University of Cambridge, Downing Street, Cambridge CB2 3EH, United Kingdom; Department of Genetics, University of Cambridge, Downing Street, Cambridge CB2 3EH, United Kingdom; Department of Computer Science, University of Oxford, Parks Road, Oxford OX1 3QG, United Kingdom

**Keywords:** pangenomics, assembly, optimization, quantum algorithms

## Abstract

*De novo* genome assembly is challenging in highly repetitive regions; however, reference-guided assemblers often suffer from bias. We propose a framework for pangenome-guided sequence assembly that can resolve short-read data in complex regions without bias towards a single reference genome. Our primary contribution is to frame the assembly as a graph traversal optimization problem, which can be implemented classically or on a quantum computer. The workflow involves first annotating pangenome graphs with estimated copy numbers for each node, then finding a path on the graph that best explains those copy numbers. On simulated data, our approach significantly reduces the number of contigs compared with *de novo* assemblers. While they introduce a small increase in inaccuracies, such as false joins, our optimization-based methods are competitive with current exhaustive search techniques. They are also designed to scale more efficiently as the problem size grows and will run effectively on future quantum computers; a small experiment on a real quantum device showcases this behaviour. Moreover, they are more resilient to noise in copy number estimation inherent in short-read-based assembly. We also develop novel tools for creating realistic synthetic pangenomes, aligning reads to pangenomes and for evaluating assembly quality.

## Introduction

A single linear reference genome alone cannot capture the genome variation that occurs within species, whether the variation is local, such as single-nucleotide variants, or larger structural changes, like copy number variations (CNVs) [1]. Bioinformatic pipelines often include a step where a new sample is compared with this fixed reference genome; e.g. in variant detection. When a single reference is used, *reference bias* is inevitable: a tendency to report fragments of sequence that appear in the reference, and to miss novel or highly divergent regions [1–3]. The solution is to use references that capture genomic diversity; one such object is the pangenome.

Pangenomics is a relatively young field, and there is no unified description of the various forms and construction methods of pangenomes. Indeed, the term “pangenome” is also applied to representations that separate the core and accessory genes present in bacterial species [4], a completely different setting than we are interested in. According to the conventions set out by Matthews *et al*. [5], we focus on *sequence-oriented pangenome graphs*, which we henceforth refer to simply as pangenomes or pangenome graphs. These objects describe the location and nature of genomic variation within a collection of individuals, most commonly from the same species. As the name suggests, they are described by mathematical graphs consisting of a set of *nodes*, representing fragments of sequence, connected by *edges*, representing fragments that are adjacent within a genome. These graphs can be created by popular tools, such as *vg* [6], *pggb* [7], and *minigraph* [8]. The Human Pangenome Reference Consortium (HPRC) recently published an initial *human* pangenome [7] containing data from 47 individuals, with a follow-up data release of >200 genomes available online.

Due to dramatic reductions in the cost of next-generation sequencing, we now have whole-genome shotgun short-read data for millions of human genomes. Using a pangenome to reconstruct the full sequences of these samples would be preferable to using standard references such as GRCh38 [9] or performing incomplete *de novo* assembly. A toolkit dedicated to providing this functionality is not currently available, though some software kits use pangenomes in other ways. *DRAGEN* [10] performs variant detection by mapping reads against a pangenome before mapping back to a comparison against a single reference genome. *Pasa* [11] uses pangenomes as a source of global information to resolve assemblies with a large number of contigs into a single sequence.

**Figure 1:**
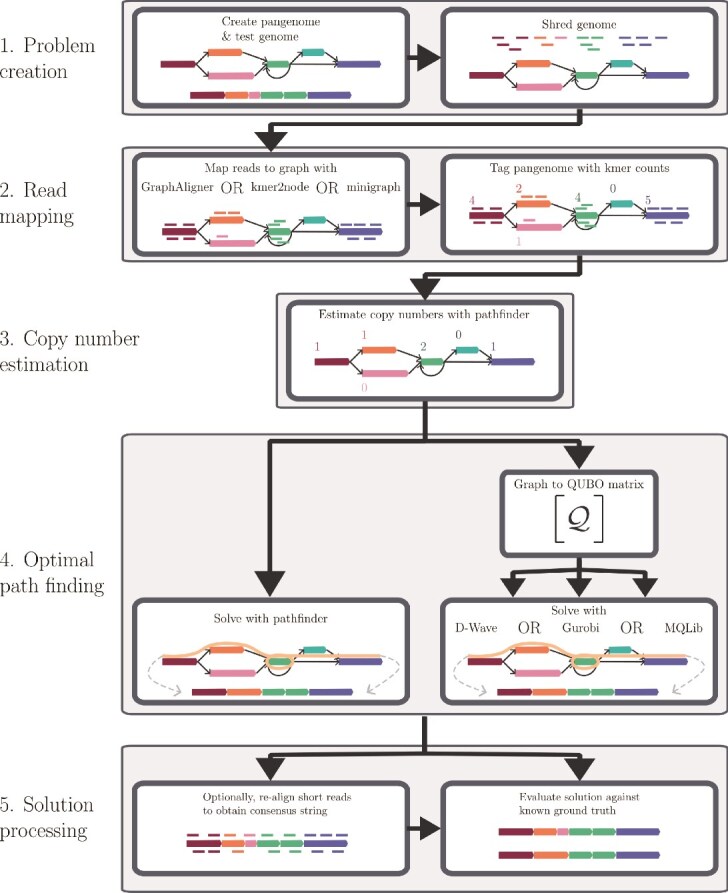
A sketch of the pangenome-guided sequence assembly procedure. (1) *Problem creation* consists of two steps. A pangenome and a new individual genome are synthesized, and the genome is shredded to simulate shotgun sequencing. (2) *Read mapping* involves aligning the short reads to the pangenome using 1 of 3 software tools and tagging the nodes with the observed kmer counts. (3) *Copy number estimation* is performed using *pathfinder*. (4) *Optimal path finding* depends on the choice of solver. If using *pathfinder*, input the annotated graph directly. Otherwise, construct the QUBO matrix from the graph, and input that into the chosen QUBO solver. (5) *Solution processing* starts with an optional re-alignment step to improve solution quality, before evaluating solution quality with a variety of metrics.

Here, we propose a methodology for *pangenome-guided sequence assembly* that maps reads onto a pangenome and reformulates the assembly as a graph-traversal optimization. Most genomic regions are easily resolved; we focus on the highly complex, repetitive regions of the pangenome graph. The pipeline, summarized in Fig. 1, consists of five stages:

Problem creation: Generate a synthetic pangenome and a new individual genome. Simulate short-read sequencing (typically 30$\times$ coverage).
Read mapping: Align short reads to the pangenome using *GraphAligner, kmer2node*, or *minigraph*, and annotate nodes with kmer counts.
Copy number estimation: Estimate node copy numbers from the annotated graph.
Path finding: Identify a path through the pangenome graph that best fits the observed copy numbers using either the *pathfinder* tool or a novel binary optimization approach.
Solution processing: Extract and optionally re-align the resulting sequence to improve quality before evaluating against the ground truth.

The key computational task in the pipeline is the path finding in step 4. This problem is similar to those tackled by certain *de novo* genome assemblers like *Flye* [12] and *Oatk* [13], which aim to resolve repeat-induced non-linear structures in assembly graphs. Their approach typically involves estimating the copy numbers of repeat sequences from sequence coverage and then identifying a graph traversal that satisfies these copy number constraints. They are designed for datasets derived from a single individual, in which the resulting graphs are typically complete and relatively noise-free, allowing the estimation of sequence copy numbers reliably.

In the context of pangenome graphs, however, additional complexities arise. First, due to genomic divergence, a pangenome graph does not fully represent the target genome, necessitating imputation of missing or divergent regions. Second, read mapping to a pangenome graph is inherently noisier due to its repetitive and multi-genome nature, resulting in greater uncertainty in sequence coverage and, consequently, in copy number estimates. To accurately resolve the graph paths through repetitive regions, pangenome-based methods must be more robust to such noise.

To that end, we formalize the path finding problem as an optimization problem. The optimization may be viewed as a generalization of the Hamiltonian path problem and is **NP**-hard in general. We cast it as a Quadratic Unconstrained Binary Optimization (QUBO), a framework commonly used in the optimization literature due to its ability to strike a balance between expressivity and tractability. Moreover, QUBO is in some sense “native” for *quantum* optimization, particularly the Quantum Adiabatic Optimization Algorithm (QAOA). Quantum computers are currently limited in size and error-prone, characteristic of the so-called Noisy Intermediate-Scale Quantum (NISQ) era. In the NISQ era, applications of quantum computers must be carefully chosen to mitigate these limitations; combinatorial optimization is a promising direction that has already seen some success [14–17]. A primer on quantum computing and quantum optimization is given in Section S1. Below, we compare both classical and quantum approaches to the optimization and discuss the potential utility of quantum approaches.

We also make several contributions that may be of interest to the broader (pan)genomics community. We develop a tool for creating synthetic pangenomes that have a realistic biological structure. The size and complexity of these pangenomes can be controlled by adjusting the rate of simulated evolutionary mutations, such as CNVs, large repeats, and translocations. We also create a novel tool *kmer2node* for aligning reads onto pangenome graphs. Finally, we develop a postprocessing pipeline to evaluate assembly quality and improve it with an optional step.

## Results

Our main comparison point for existing state of the art is *pathfinder* [13]. *Pathfinder* is a submodule from the *Oatk* package, a tool for resolving plant organelles using high-accuracy long reads. While this application is far from our current setting, the methods are somewhat related. In particular, *pathfinder* also assigns weights to a graph and then searches for a walk that explains the data. However, it proceeds via exhaustive search rather than optimization; moreover, *pathfinder* was designed to resolve assembly graphs whose annotated coverage values are essentially noiseless and have no unused nodes. Conversely, in our setting, the reads originate from an individual *not* present in the pangenome, and we therefore expect imperfect coverage values and many nodes to have zero coverage. We use a cost function to be robust to this noise.

As an example, consider a simple line graph with nodes $A \rightarrow B \rightarrow C \rightarrow D \rightarrow E$ and estimated copy numbers $(1,\, 1,\, 0,\, 1,\, 1)$. The prescriptive nature of *pathfinder* yields two contigs: $A \rightarrow B$ and $C \rightarrow D$. Conversely, the cost function used in our QUBO formulation allows a single unified path of $A \rightarrow \ldots \rightarrow E$ to be found. Nevertheless, to the best of our knowledge, *pathfinder* is the software package that gives the fairest comparison with our results.

A comparison of pangenome-guided assembly of synthetic haploid data via kmer mapping using *pathfinder* against *de novo* sequence assembly can be seen in Table 1. Weight assignment methods are discussed in detail in section S3. Averaged stats from 51 seeds with 5 alignments each. *Pathfinder* results are presented twice, evaluating the original concatenated path and an optimized path formed by realigning all the short-read data back to the original path to produce a refined consensus sequence. Columns represent the proportion of the true genome covered by the assembly result (%Covered), and the percentage of this result covered by the true genome (%Used), the number of contigs, the number of breaks in alignment of assembly to true sequence (breaks, i.e. false joins), the number of large indels and regions of significant runs of differences, the N50 contig size, and total percent identity between assembly and the true genome. Best performers are shown in bold. See Section “Classical postprocessing: evaluating path solutions” for further details. The primary difference lies in the number of contigs returned and demonstrates the great benefit of using a pangenome-guided approach to short-read assembly. However, this comes with a small increase in false joins (i.e. breaks in alignment with the truth set). Optimizing the path sequence by realigning the short-read data against it and producing a new consensus improves accuracy across a range of metrics. The percentage identity remains slightly lower than that of the *de novo* assemblers, indicating that a hybrid solution may be the optimal strategy.

**Table 1:** Performance of pangenome guided mapping of short-read data using *minigraph, GraphAligner, vg giraffe*, and *kmer2node* weight assignments with the *pathfinder* solver, compared against pure *de novo* sequence assembly from *syncasm* and *miniasm*.

**Annotator/Assembler**	**%Covered**	**%Used**	**Contigs**	**Breaks**	**Indel**	**No.Diff**	**N50**	**%Identity**
Kmer2node	88.3	93.2	2.8	1.9	1.2	0.2	6697	96.9
Minigraph	85.1	91.2	4.7	2.3	1.0	**0.0**	5044	97.2
GraphAligner	92.6	92.0	1.7	2.0	1.7	0.2	9484	96.9
Giraffe	91.7	91.7	1.9	2.0	1.4	0.1	8748	97.0
Kmer2node-opt	88.4	**94.0**	2.6	1.5	0.6	0.3	6679	98.7
Minigraph-opt	85.4	92.9	4.3	1.8	0.3	0.1	5002	99.2
GraphAligner-opt	**93.0**	92.6	**1.6**	1.6	0.8	0.3	**9436**	98.6
Giraffe-opt	91.9	92.6	1.8	1.7	0.7	0.3	8701	98.7
Syncasm	91.6	86.2	31.3	**0.0**	**0.1**	**0.0**	878	**100.0**
Miniasm	81.5	91.3	14.8	0.1	**0.1**	**0.0**	1022	99.6

To evaluate the optimization formulation, we tested a range of solvers with varying levels of quantum integration. These methods span a spectrum from traditional classical solvers, to hybrid approaches like quantum annealing QA, which is specialized for optimization problems, to gate-based quantum computers.

Classical solvers and quantum annealers can accept large problems with up to several thousand variables. However, they do not necessarily find optimal or even good solutions within a reasonable time frame at this scale. Conversely, the quantum circuit approach is very limited in scope. Quantum optimization experiments have been run on hardware with up to 127 qubits [17]. In this work, we limit ourselves to simulations of such experiments. Due to the exponentially scaling cost of simulating quantum systems as the number of qubits increases, we only consider problems with up to 35 qubits. The results of these simulations serve as a proof-of-principle for a quantum approach as hardware size and quality improve in the coming years.

### Classical optimizers

We first benchmark the performance of our formulation using two classical optimization suites. *Gurobi* [18] is an industry-standard package that employs branch-and-bound methods to find provably optimal solutions to a range of optimization problems, including mixed-integer quadratic programming tasks which admit QUBO as a special case. *MQLib* [19] is a package that provides fast implementations of several heuristics for QUBO. We use the multistart tabu search strategy introduced by Palubeckis [20] since it performs most consistently on our instances.

We test the performance of each solver—*pathfinder, Gurobi,* and *MQLib*—on graphs obtained with each annotation strategy—*GraphAligner, minigraph,* and the in-house *kmer2node* tool. We use the oriented tangle resolution problem, defined in Section “Formulating sequence assembly as an optimization problem”, as it captures the biological nature of the task while having reduced complexity compared with the Diploid version, making it easier to benchmark across all the solution strategies.

**Figure 2:**
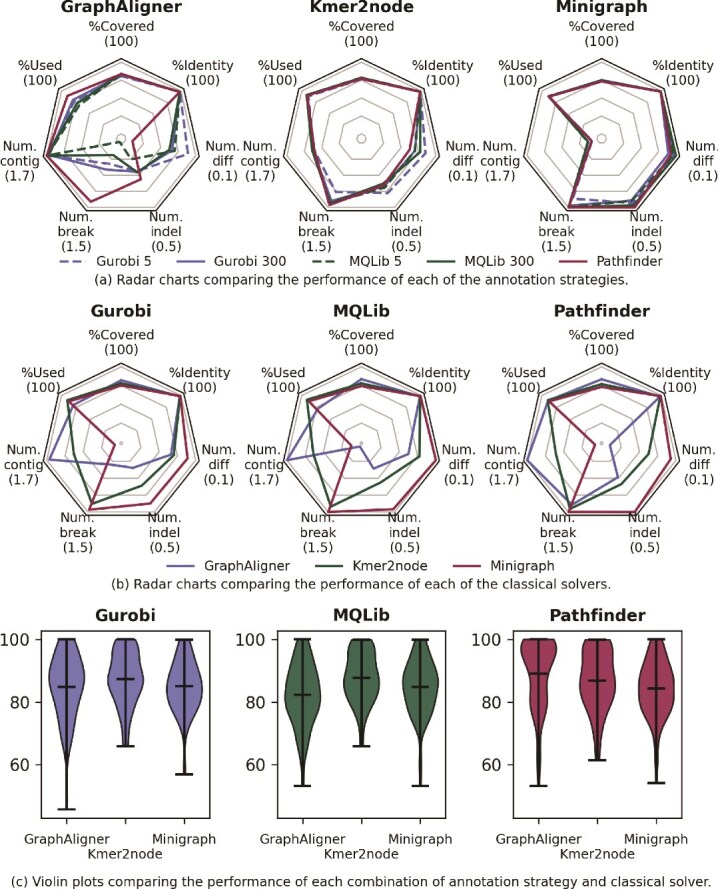
(a) and (b) Radar charts comparing the performance of each combination of annotation strategy: *GraphAligner, kmer2node*, and *minigraph*; and classical solver: *Gurobi, MQLib*, and *pathfinder*. The seven axes plotted are the evaluation criteria discussed in Section “Classical postprocessing: evaluating path solutions”. For each, the number in brackets corresponds to the outermost point on that axis. For the lower half of each plot, further out along the axis corresponds to fewer contigs, breaks, and so on. (c) Violin plots showing the per-instance performance of *Gurobi, MQLib*, and *pathfinder*.

We test each combination on a set of 20 pangenomes with 5 sequences to be aligned against each pangenome. The pangenomes have an average of 77.8 nodes. *Gurobi* and *MQLib* are given time limits of 5 and 300 s to test whether higher quality solutions are obtained at long run times. These classical optimizers are allowed 3 runs per sequence and per time limit to account for variations in solution quality due to their heuristic nature. The averaged results are given in Table 2, with standard deviations given in parentheses and best performers shown in bold. Radar charts showing the average performance across the metrics discussed in Section “Classical postprocessing: evaluating path solutions” are given in Fig. 2. Figure 2a shows that, for any choice of annotation strategy, each of the solvers is broadly competitive with the others. When either *kmer2node* or *minigraph* is used, the average case results across each of the solvers are very similar. The starkest difference comes when *GraphAligner* is used. In that case, *pathfinder* reports more large differences than either *MQLib* or *Gurobi*, but fewer breaks and slightly fewer large indels on average. The large number of breaks in the QUBO solver results may be due to not taking into account edge weights, which can help to resolve inversions: see Section “Discussion” for a brief discussion.

**Table 2:** Average performance of pangenome guided mapping of short-read data using *minigraph, GraphAligner,* and *kmer2node* weight assignments with the *MQLib, Gurobi,* and *pathfinder* solvers.

**Annotator**	**Solver**	**%Cov**.	**%Used**	**Contigs**	**N50**
GraphAligner	Gurobi	85.69 (12.46)	83.85 (11.75)	1.92 (1.52)	**9755** (2699)
	MQLib	**86.90** (12.97)	77.75 (13.60)	**1.73** (1.13)	11337 (3846)
	Pathfinder	86.74 (15.82)	91.42 (8.56)	**1.73** (1.13)	8725 (2194)
Kmer2node	Gurobi	82.50 (12.52)	92.07 (7.68)	3.73 (2.50)	5370 (2529)
	MQLib	82.36 (12.19)	**92.88** (6.44)	3.68 (2.27)	5201 (2523)
	Pathfinder	81.55 (13.42)	92.01 (7.76)	3.85 (2.71)	5208 (2586)
Minigraph	Gurobi	80.09 (11.74)	89.92 (7.37)	6.74 (3.69)	3512 (2708)
	MQLib	79.40 (13.45)	90.31 (7.59)	6.47 (3.58)	3468 (2696)
	Pathfinder	78.51 (13.84)	90.06 (7.48)	6.74 (3.84)	3409 (2684)
**Annotator**	**Solver**	**Breaks**	**Indels**	**Diffs**	**%Identity**
GraphAligner	Gurobi	5.31 (3.76)	1.09 (1.09)	0.18 (0.48)	98.45 (1.38)
	MQLib	6.76 (4.90)	1.08 (1.14)	0.19 (0.44)	98.42 (1.35)
	Pathfinder	2.09 (2.08)	0.97 (1.18)	0.33 (0.62)	98.21 (1.90)
Kmer2node	Gurobi	2.17 (2.09)	0.86 (1.04)	0.17 (0.40)	98.82 (1.14)
	MQLib	1.95 (1.72)	0.90 (1.14)	0.15 (0.36)	98.75 (1.32)
	Pathfinder	1.76 (1.53)	0.88 (1.05)	0.19 (0.42)	98.70 (1.34)
Minigraph	Gurobi	1.70 (1.58)	0.63 (0.95)	0.12 (0.35)	99.09 (0.93)
	MQLib	**1.52** (1.40)	0.56 (0.82)	**0.09** (0.29)	99.14 (0.75)
	Pathfinder	**1.52** (1.34)	**0.52** (0.79)	0.11 (0.34)	**99.17** (0.91)

As well as average-case performance, it is illuminating to see how the combinations perform on specific instances, particularly hard ones. Violin plots showing the average of the “Covered” and “Used” statistics across all instances are given in Fig. 2c. When *GraphAligner* is used to annotate graphs, it is clear that *pathfinder* outputs high-quality solutions on a majority of instances, although a very low worst-case outcome drags down its average performance. For the other annotators, the QUBO solvers are much more competitive, with similarly shaped plots across both the *minigraph* and *kmer2node* settings.

### Quantum annealing

To test the potential of QA, we perform a range of experiments using the *Leap* hybrid solver provided by the quantum hardware company *D-Wave* [21]. This solver is provided on the cloud, and typically one or more classical algorithms run on the problem while hard parts are outsourced to the QPU. A configurable time limit is provided, and the best solution found during that time is returned, along with some metadata. The main benefit of QA is the size of the hardware available. The Advantage2 system has 4400 qubits arranged in a lattice structure, $\sim$30 times larger than superconducting offerings from IBM, and $\sim$90 times larger than Quantinuum’s trapped ion computers. We use the QA systems to wayfind the potential for quantum advantage on more standard quantum hardware in the future.

**Figure 3:**
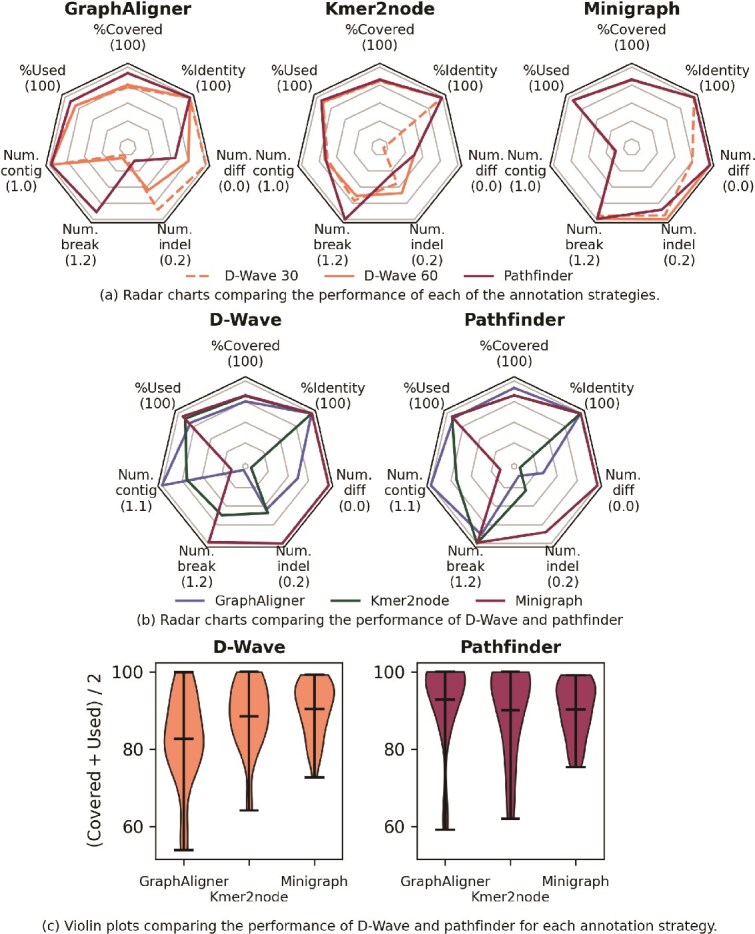
(a) and (b) Radar charts comparing the performance of each combination of annotation strategy: *GraphAligner, kmer2node*, and *minigraph*; with quantum annealing solver *D-Wave* or classical solver *pathfinder*. The seven axes plotted are the evaluation criteria discussed in Section “Classical postprocessing: evaluating path solutions”. For each, the number in brackets corresponds to the outermost point on that axis. For the lower half of each plot, further out along the axis corresponds to fewer contigs, breaks, and so on. (c) Violin plots showing the per-instance performance of *D-Wave* and *pathfinder*.

We test *D-Wave* on 10 pangenomes, with 3 sequences to be aligned to each. Due to limited access to *D-Wave* systems, we tested on smaller instances with an average of 44.4 nodes per graph. The annealer was allowed 2 runs per sequence, with a time limit of either 30 or 60 s. Results are given in Table 3, with best performers shown in bold, and visualized in Fig. 3. For the instances tested, there is a clear benefit to using *minigraph* as an annotator; in that case, *D-Wave* performs comparably well with *pathfinder*.

**Table 3:** Average performance of pangenome guided mapping of short-read data using *minigraph, GraphAligner*, and *kmer2node* weight assignments with the *D-Wave* and *pathfinder* solvers.

**Annotator**	**Solver**	**%Cov.**	**%Used**	**Contigs**	**N50**
GraphAligner	D-Wave	80.18 (13.25)	85.07 (13.49)	**1.10** (0.54)	9062 (1727)
	pathfinder	**93.24** (11.76)	92.29 (11.00)	**1.10** (0.54)	**10 060** (385)
kmer2node	D-Wave	85.45 (11.31)	91.52 (8.55)	1.97 (1.60)	7203 (2315)
	pathfinder	85.99 (16.44)	93.99 (8.65)	2.00 (1.59)	7330 (2586)
minigraph	D-Wave	85.99 (10.85)	**94.75** (6.37)	3.53 (1.71)	4361 (1879)
	pathfinder	86.03 (11.15)	94.43 (6.93)	3.53 (1.71)	4492 (1913)
**Annotator**	**Solver**	**Breaks**	**Indels**	**Diffs**	**%Identity**
GraphAligner	D-Wave	5.77 (4.26)	0.53 (0.96)	0.07 (0.25)	99.18 (0.98)
	pathfinder	1.77 (2.73)	0.83 (1.00)	0.10 (0.30)	99.22 (1.15)
kmer2node	D-Wave	2.97 (2.34)	0.50 (0.76)	0.13 (0.34)	99.08 (1.13)
	pathfinder	**1.23** (1.52)	0.70 (0.90)	0.13 (0.34)	99.14 (1.03)
minigraph	D-Wave	1.30 (1.07)	**0.23** (0.50)	**0.03** (0.18)	99.55 (0.53)
	pathfinder	1.27 (1.26)	0.33 (0.70)	**0.03** (0.18)	**99.58** (0.53)

### Quantum optimization

We also test hybrid classical-quantum optimization algorithms as a proof of concept. We use various techniques to improve the convergence of the algorithm—see Section “Classical simulation of QAOA” for details. For the purposes of testing our algorithms, the majority of runs are performed with the quantum computer being simulated classically. We can simulate circuits up to 35 qubits using heterogeneous CPU + GPU simulations. However, we also perform small experiments on real quantum devices; a comparison of simulated and actual performance for one such experiment is provided below.

**Figure 4:**
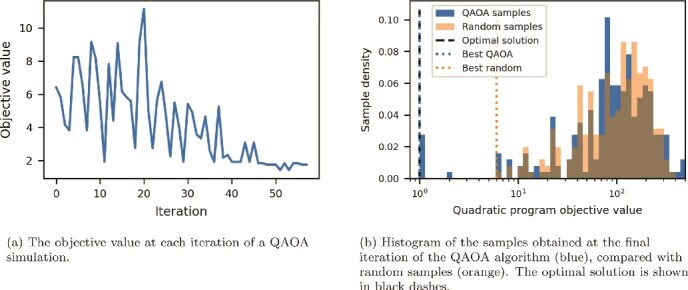
Plots showing the performance of a 10-qubit QAOA simulation for oriented tangle resolution.

Figure 4 shows the results of simulating the QAOA algorithm with $p = 4$ on a small oriented tangle resolution problem. The circuit has 10 qubits, 496 2-qubit gates and a 2-qubit depth of 112 after being compiled to the IBM-native gate set. The chosen emulator implements noiseless gates, but we include sampling noise: 256 samples are taken at each iteration. A maximum of 100 iterations were allowed. The simulation took under 20 s to run on a single CPU and GPU, including circuit compilation time. Figure 4 demonstrates the successful convergence of the QAOA procedure. The simulated quantum computer sampled the optimum repeatedly and had several other samples at the same energy as the best random samples.

**Figure 5:**
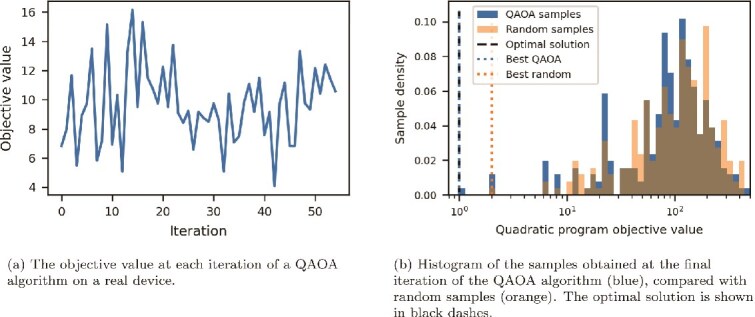
Plots showing the performance of a 10-qubit QAOA simulation for oriented tangle resolution.

Figure 5 shows the results of running the same QAOA algorithm, with the same initial conditions, on an actual quantum device. The device used is the *IBM Strasbourg* computer, an Eagle R3 device with 127 qubits in a “heavy-hex“ layout and an average 2-qubit error rate of around $3 \times 10^{-2}$. The full computation took 213 s. Each of the 55 iterations of the algorithm used $<1$ s of QPU time; the remaining time is comprised of classical computation, communication with the device, and then preparing the device for the next iteration. Clearly, noise in the device has degraded the convergence of the classical optimizer. Nonetheless, Fig. 5b still shows a non-trivial shift of probability mass towards lower-energy solutions. Moreover, the optimal solution was again sampled, and several other low-energy solutions were found.

Going forward, it is clear that error mitigation techniques are critical for the QAOA algorithm to succeed. Since we are essentially considering a sampling task, measurement error mitigation will likely be a good choice. The implementation of these techniques is left for future work.

## Discussion

In this work, we initiate a study on using pangenomes to aid in the assembly of short-read data. We adopt a novel optimization approach to tackle assembly, which we believe is well suited to mitigate the noise inherent in short-read sampling. We demonstrate that our methods enhance the accuracy of the assembly while significantly reducing the number of contigs reported compared with state-of-the-art *de novo* assemblers. The optimization is designed to be amenable to both classical and quantum computing approaches and scales more favourably with problem size than existing methods.

Moreover, in challenging cases such as repetitive regions, our approach is more robust since errors during the copy number estimation step will be less impactful on our cost function approach compared with the existing state-of-the-art *pathfinder*, whose copy number assignment is prescriptive. For such instances, all sequence assembly methods will suffer from decreased accuracy due to the difficulty of assigning kmer counts; our *kmer2node* tool includes mitigation strategies, which we discuss in section S3.2.

Our pipeline consists of three main stages: graph annotation, copy number estimation, and path finding. We investigate the impact on solution quality of using different software for both the graph annotation and path finding steps. For copy number estimation, we only use the capabilities of *pathfinder*. Further exploration in this direction could certainly be fruitful; one option might include training a machine-learning model to perform this task.

For the graph annotation, we find that *minigraph* generally performs strongly, minimizing the number of breaks in the assembly as well as the number of large indels, at the cost of reporting several contigs per assembly. We usually prefer several accurate contigs over fewer, more inaccurate ones.

For the solvers, we see that our (classical) binary optimization approaches are at the very least competitive with the bleeding-edge bioinformatics tool *pathfinder*. On the intermediate-size instances we test, the classical optimizers report good results within reasonably short time limits, often reporting better solutions than *pathfinder* on specific problems. They could be included as a parallel assembly method when high-quality results are desired, taking a consensus of all the candidate solutions as the final answer. As the problem size increases and the exhaustive search of *pathfinder* is no longer feasible, optimization methods may be better placed to report reasonable assemblies.

We also investigate the feasibility of quantum computing in solving these problems. We first test a QA approach on a set of middling-complexity problems, observing that the solution quality is broadly similar to *pathfinder*, even though only short time limits were used. This is a positive sign for the potential of using QA to solve industry-scale problems in the future, as hardware continues to improve. However, there is currently no evidence of utility in using these systems for our problem.

Finally, we simulate the QAOA on some small problems as an initial indicator of the potential for standard, circuit-model quantum computers to solve pangenomic problems. We can converge a QAOA experiment at this small size. While performing a hardware experiment at an industry scale is required to make any serious claims about quantum utility in this domain, we see this as a promising proof of principle.

For a discussion of possible future research directions, see section S2.

## Methods

### Problem inputs

Our primary input is unaligned short sequences of genomic data. We have sufficient coverage of this to sequence every base in the target genomic region to a sufficient depth (typically at least 30$\times$). We also have a previously computed pangenome in Graphical Fragment Assembly (GFA) format, with DNA sequence fragments in graph nodes, and edges linking nodes together to describe paths through the graph corresponding to the training data. The unaligned short sequences are then used to annotate this GFA with node and edge weights, where the weights indicate the expected number of traversals through those graph elements. The problem is then how to identify the optimal path through the weighted graph i.e. most consistent with the specified weights.

In order to assess the accuracy of our methods, we use synthetic haploid data so we have a known ground truth. We generate a population of 100 related genomes, iterated over 10 generations. A random subset of 40 members of this population is used to build a pangenome, while a further 10 different genomes are used to evaluate our methods by simulating whole-genome shotgun experiments from a short-read sequencing instrument. These short fragments are mapped back to the graph, producing the weighted GFA.

### Pangenome creation

Tools such as *minigraph* prefer to build a pangenome, i.e. broadly co-linear with the input genomes. This reduces the number of tangles and loops in the graph, but it means repeated sequence may end up in multiple nodes. This is the style of pangenome published by the HPRC, which has the benefit of allowing standard linear reference coordinates, such as GRCh38, to be threaded through the pangenome graph. Therefore, this is the style of pangenome graph we focus on.

A pangenome can also be complete or sparse: it could include every single base difference used in the input data, which lead to a large graph with many nodes and edges, or it could collapse small localized variations into a single node. In this case, the node sequence will be either a representative single sequence or a consensus of all the donor genomes that passed through that node during graph construction. *Minigraph* takes this latter approach, which is useful since we need to keep the graph complexity low for the problem size to be amenable to existing quantum computers.

We build a simulated genome population using our own tool *genome_create* with fixed random seeds for reproducibility. The initial genome contains Short-Tandem Repeats, longer CNVs, short and long repeat elements, translocations and inversions, and random point mutations. Each subsequent population member is haploid, being derived from a single randomly chosen previous genome. This is then further modified by the same types of variation listed above, but at a substantially reduced rate, giving rise to a population of similar sequences with a common complex structure that can form population graphs. We use a subset of our population to build the pangenome graph using *minigraph*. The remaining sequences are used to simulate shotgun sequencing by replicating to 30-fold coverage and fragmenting into short sequences, with a uniform distribution of sequencing errors.

### Annotating the graph with copy numbers

For each member of the training set, we produce fake short-read sequencing data. For simplicity, this is single-ended (i.e. not in pairs from a larger template). They are randomly distributed from either strand with a random distribution of base-substitution errors; no indel errors are modelled.

Existing tools that take a short sequence, align it against a graph (GFA) and record the path include *GraphAligner, vg giraffe*, and *minigraph*. We also produce our own *kmer2node* tool. All four tools annotate the GFA with edge-traversal counts and kmer counts within node sequences. The presence of repeats can still be problematic, as multiple paths may exist. It may not be possible to distinguish genuine sequence rearrangements from secondary hits arising from multiple copies of the repeated sequence in the pangenome. When reported, we filter out secondary alignments.

As mentioned earlier, our pangenome graph node sequences are a single representative or consensus sequence. However, the training genomes used to construct that pangenome may have many point mutations and small indels, which reduce kmer match rates. *Kmer2node* may use a list of recognition sequences, so we align the full-length training genomes to the pangenome using *GraphAligner* to produce a list of recognition sequences per node instead. This improves our kmer hit rate, but may also decrease the percentage that uniquely map. While indexing the GFA, we also get an estimate of the percentage of kmers we expect to be unique. This provides us with a kmer hit-rate expectation during alignment of short-read data, allowing us to improve the assessment of node sequence depth.

All the tools produce a GFA with kmer counts per node and frequencies of edge traversals. For *pathfinder*, this is sufficient as it can convert these to depth-normalized counts. To aid consistency, we also use *pathfinder’s* subgraph connection analysis and depth-normalized counts as input to the QUBO solvers.

### Classical postprocessing: evaluating path solutions

Our path solvers produce a list of nodes and their orientation. From this, we can concatenate the sequences recorded in the GFA nodes, reverse-complementing as necessary, to produce a candidate genome sequence. Note that even if the path is perfect, this candidate genome may not be identical to the true genome, as our GFA files produced by *minigraph* collapse small variations into a single node. It is also possible that the true genome has novel structural rearrangements (copy-number changes or translocations) not present in the training data used to produce the pangenome graph, so a perfect path may not exist.

We align this candidate genome sequence against the known truth using *bwa mem*, reporting only primary and supplementary hits. This produces one or more alignments, each representing co-linear matches between the candidate and true genomes. The alignments are broadly equivalent to the contigs produced by a *de novo* assembler. Each alignment record may, in turn, have smaller variations (substitutions and small indels) that are not large enough to split the alignment into two. This provides a unified framework for comparing pangenome path reconstruction with more traditional *de novo* sequence assembly tools. We report the number of contigs and their N50 size. We align the contigs to the true genome to estimate the proportion of the true genome we have sampled, and vice versa. This two-way approach enables us to detect both over- and under-calling of copy-number variants.

These provide basic evidence on the contiguity of the assembled data, but not its accuracy. To this end, we also report the number of breaks when aligning these contigs back to the true genome (indicating incorrect path forks or large indels that require a supplementary alignment). These are analogous to false joins from a *de novo* assembler. For finer-grained evaluation, we also report small indels ($\geq$ 10 bp), regions of substantial base variation (multi-nucleotide polymorphism of 30% variance within a 100 bp window), and the overall percentage identity. Although BUSCO metrics are frequently used to assess assembly quality, these experiments assemble only small regions of synthetic genomes that do not contain any BUSCO genes, so it is not possible to use BUSCO for theevaluation in this case.

We expect most users to prefer more contigs over incorrectly joined contigs, but the relative weighting of the two is a subjective decision that will depend on downstream usage.

A further optional refinement step is to realign the short reads to the generated candidate genome sequence and then take the consensus of the newly aligned sequences. This corrects for novel mutations in this sample or simplifications made during the *minigraph* pangenome creation step.

### Formulating sequence assembly as an optimization problem

After annotation, our data are a graph with non-negative weights on the nodes and edges. Our current formulation only uses node weights, whereas tools such as *pathfinder* can use both sets of weights. Extending our methodology to include edge weights is a future direction that we discuss in section S2.

We formally define the pangenome graph annotated with copy numbers as a triplet $G = (V, E, w)$, where $V$ is the set of vertices, $E \subseteq V \times V$ is the set of edges, and $w: V \rightarrow \mathbb{R}_{+}$ is a weight function giving the estimated copy number of each vertex.

We then seek a walk on the graph that best explains the copy number data. This graph-traversal problem shares superficial similarities with the Travelling Salesman Problem (TSP). In particular, we define the following optimization problem, which we refer to as *Tangle Resolution* due to the often complex, knot-like structures present in pangenomes. Note the implicit requirement in the problem that each step in $W$ traverses an edge in $G$.

Problem 1 (Tangle Resolution). Given a vertex-weighted graph $G = (V, E, w)$, find a walk $W$ on $G$ that minimizes

1 \begin{eqnarray*}& C_{G}(W) = \sum_{v \in V} \Bigl( \#W(v) - w(v) \Bigr)^{2},\end{eqnarray*}

where $\#W(v)$ is the number of times $W$ visits $v$.

While this model serves as a useful starting point, it does not encompass all the biological features. Most notably, it fails to account for the double-stranded nature of genomic data. To better reflect the biology, we must consider a *directed* graph. The vertex set is now formed of two halves $V_{+}, V_{-}$. The sequence data corresponding to $v_{-} \in V_{-}$ are the reverse complement of the sequence data corresponding to $v_{+} \in V_{+}$. Edges of the graph are directed and come in pairs: an edge $A+ \rightarrow B+$ implies the existence of an edge $B- \rightarrow A-$, and vice versa. We call such a graph $G = (V_{+}\cup V_{-}, E, w)$  *oriented*.

The corresponding optimization task is similar, but we now sum the visits over both the positive and negative orientations. This is captured in the following problem statement, which we refer to as *oriented tangle resolution*.

Problem 2 (Oriented tangle resolution). Given a vertex-weighted, oriented graph $G = (V_{+}\cup V_{-}, E, w)$, find a walk $W$ on $G$ that minimizes

2 \begin{eqnarray*}& C_{G}(W) = \sum_{v \in V} \Bigl( \#W(v_{+}) + \#W(v_{-}) - w(v) \Bigr)^{2},\end{eqnarray*}

where $\#W(v_{+})$ and $\#W(v_{-})$ are the number of times $W$ visits $v_{+}$ and $v_{-}$, respectively.

Finally, for the setting of human data, we need to resolve diploid DNA. We therefore need to find a *pair* of walks $W_{1}$ and $W_{2}$ through an oriented graph that combine to explain the data. Formally, we have *diploid tangle resolution*:

Problem 3 (Diploid tangle resolution). Given a vertex-weighted, oriented graph $G = (V_{+}\cup V_{-}, E, w)$, find a pair of walks $W_{1}$ and $W_{2}$ on $G$ that minimize

3 \begin{eqnarray*}& C_{G}(W_{1}, W_{2}) = \sum_{v \in V} \left( \sum_{i=1}^{2} \Bigl( \#{W_{i}}(v_{+}) + \#{W_{i}}(v_{-}) \Bigr) - w(v) \right)^{2},\end{eqnarray*}

where $\#W_{i}(v+)$ and $\#W_{i}(v_{-})$ are the number of times walk $W_{i}$ visits $v_{+}$ and $v_{-}$, respectively.

This formulation naturally extends to polyploid data by increasing the number of walks; we will not consider that setting here.

### Mapping guided alignment to binary optimization

The QUBO formulation defines a class of optimization problems characterized by the minimization of a quadratic polynomial over binary variables ${x_{i}}$. Since $x_{i}^{2} = x_{i}$ for $x_{i} \in \{0,1\}$, any linear term can be replaced by a quadratic one. Also, any constant offset can be neglected since it just shifts the energy landscape without affecting the optimal assignment. Therefore, the objective can be formally defined as minimizing $C(\{x_{i}\}) = x^{T}Mx$, where $x\in \{0,1\}^{n}$ and $M\in \mathbb{R}^{n \times n}$ is a symmetric, real-valued square matrix. For the sake of clarity, we express our cost functions here as standard polynomials, without reducing to a matrix form. QUBO problems enjoy a high degree of expressivity, being able to represent classic combinatorial optimization problems such as MAX-CUT and the TSP, while also remaining tractable to modern solvers at moderate sizes. Solution techniques include generic branch-and-bound methods, as employed by commercial solvers such as *Gurobi*, as well as domain-specific heuristics used by the open source library *MQLib*.

QUBO is equivalent, under a linear change of variables, to the Ising model in statistical physics, a formulation extensively used in quantum annealing devices such as those developed by *D-Wave* Systems. As such, QUBO is widely employed in quantum computing contexts, where its unconstrained nature and binary domain align with the hardware capabilities of current quantum and neuromorphic processors. The primary advantages of QUBO include its flexibility in encoding diverse problems within a unified mathematical framework and its compatibility with both quantum and classical heuristic solvers. Its unconstrained nature simplifies solver implementation by eliminating the need for explicit constraint handling, instead requiring constraint satisfaction to be incorporated into the objective function through penalty terms.

Notably, however, the QUBO forms of many classic graph-traversal problems, such as the TSP, require quadratically many variables in the graph size, with each variable encoding whether the path visits a certain node at a certain time. QUBO formulations of these tasks traditionally use binary variables $x$ with two indices $t$ and $v$, with the variable $x_{t,v} = 1$ if the solution path visits vertex $v$ at time $t$. The cost function is easily formulated by summing the cost of travelling between any two vertices $v$ and $v^{\prime}$, $c_{vv^{\prime}}$, over the edges that are traversed. This leads to an objective term $\sum _{t, v, v^{\prime}} c_{vv^{\prime}} \cdot x_{t,v}x_{t+1,v^{\prime}}$. For an assignment to represent a valid path, it is necessary to impose the constraint that exactly one vertex is visited at each time. This is accomplished through adding Lagrange multiplier terms to the cost function for each $t$, taking the form $\bigl (\sum _{v \in V} x_{t,v}- 1\bigr )^{2}$.

We propose a QUBO formulation for each of the three optimization problems, problems 1–3. For concreteness, we focus here on the simplest, problem 1.

**Figure 6:**
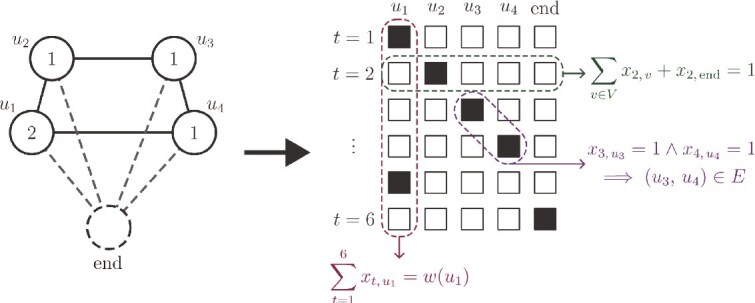
A sketch of the procedure for converting a graph-traversal problem to a QUBO problem. (Left) A graph annotated with weights, corresponding to a pangenome with reads aligned. Also shown is the virtual “end” node used in the QUBO formulation. (Right) A diagrammatic representation of the QUBO, with binary variables $x_{t,\,v}$ shown as squares that are filled if $x_{t,\,v}=1$. Highlighted are some of the constraints that we impose: upper-right, the constraint that a single variable is “on” at time 2; lower-right, that the path traverses a graph step going from time 3 to 4; bottom, that the path visits vertex $u_{1}$ twice, according to its weight.

Similarly to QUBO forms of the TSP, our formulation uses quadratically many variables. In particular, our formulation uses $(N+1)T$ variables, where $N = |V|$ is the number of vertices in the graph, and $T$ is the maximum length of a walk we are willing to consider. In practice, we choose $T = \alpha \sum _{v \in V} w(v)$ for some constant $\alpha> 1$, which we usually take equal to $1.2$. This allows walks to visit some vertices more than the data suggest, if doing so significantly improves the quality of another part of the solution. The extra vertex introduced is a virtual “end” node with no sequence data attached. A walk can spend several consecutive time steps in the end node without incurring any cost, allowing walks to effectively finish early. However, any step that leaves the end node is heavily penalized. A sketch of this process is given in Fig. 6.

The total cost function consists of three parts: the first is a Lagrange multiplier term to enforce that the variable assignment encodes a walk that visits exactly one vertex at each time; the second is a multiplier term to enforce that, at each step, the walk traverses a graph edge, including travelling to or remaining in the virtual “end” node; and the final term encodes the cost function $C_{G}(W)$.

In particular, we write the QUBO cost function as $C_{G}(\{x_{t,v}\}) = C^{1}_{G}(\{x_{t,v}\}) + C^{2}_{G}(\{x_{t,v}\}) + C^{3}_{G}(\{x_{t,v}\})$, where


4 \begin{eqnarray*} C^{1}_{G}(\{x_{t,v}\}) &= \Lambda_{1} \sum_{t=1}^{T} \left( \sum_{v \in V}x_{t,v} + x_{t,\textrm{end}}-1\right)^{2},\end{eqnarray*}

5 \begin{eqnarray*} C^{2}_{G}(\{x_{t,v}\}) &= \Lambda_{2} \sum_{t=1}^{T-1}\left( \sum_{v,v^{\prime}in V}x_{t,v}x_{t+1,v^{\prime}} \mathbb{1}_{\{(v,v^{\prime}) \notin E \}} + \sum_{v \in V}x_{t,\textrm{end}}x_{t+1,v} \right), \end{eqnarray*}

6 \begin{eqnarray*}C^{3}_{G}(\{x_{t,v}\}) &= \sum_{v \in V} \left( \sum_{t=1}^{T} x_{t,v} - w(v) \right)^{2}.\end{eqnarray*}

There is some art to choosing the values of the Lagrange multipliers $\Lambda _{1},\Lambda _{2}$. If they are sufficiently large, then we can guarantee that the assignment that minimizes $C_{G}(\{x_{t,v}\})$ encodes a walk that minimizes $C_{G}(W)$. However, if they are too large, then the energy landscape will have steep valleys around assignments that satisfy the constraints, and changes in the cost function will be nearly indistinguishable to the optimizer. We find that intermediate-size values of $\Lambda _{1} = 10$ and $\Lambda _{2} = 5$ work well, in practice. Empirically, for instances where copy number estimation was accurate, the penalties are small enough that the $C^{3}_{G}$ part is significant enough for the optimizer to find the true solution. Conversely, when copy number estimation is especially noisy, the penalties force a solution that obeys the constraints imposed by $C^{1}_{G}$ and $C^{2}_{G}$, overriding the inaccurate constraints of $C^{3}_{G}$.

To extend this method to oriented tangle resolution, we simply introduce a pair of variables for each vertex and each time, resulting in $(2N+1)T$ variables. For diploid tangle resolution, we further introduce a new set of variables $y_{t,v}$ for the second path, resulting in $2(2N+1)T$ variables. These variables have independent copies of $C^{1}_{G}$ and $C^{2}_{G}$ to enforce the constraints, but share a combined form of $C^{3}_{G}$.

### Mapping quadratic unconstrained binary optimization problems to quantum optimization

Quantum optimization, especially for combinatorial optimization problems, is a promising application of near-term quantum computers [14–16]. One particularly fruitful technique is the Quantum Approximate Optimization Algorithm (QAOA) [22–25]. In this setting, the cost function of the optimization problem is cast to a quantum Hamiltonian, whose ground state encodes the optimal solution to the problem.

In the case of QUBO problems, this conversion is particularly simple. The Hamiltonian $H$ has as many qubits as there are binary variables in the QUBO. To obtain $H$, we replace each binary variable $x_{i}$ in the cost function $C$ with a quantum operator $(I-Z_{i})/2$, where $Z_{i}$ is the Pauli-$Z$ gate acting on the $i{\textrm{th}}$ qubit. $H$ is then a sum over identity, $Z$ and $ZZ$ terms. Since $Z\ket{0} = \ket{0}$ and $Z\ket{1} = -\ket{1}$, we see that $\tfrac{1}{2}(I-Z)\ket{b} = b\ket{b}$ for $b \in \{0,1\}$. Therefore, the *energy* of a state $\ket{x}$, given by $\bra{x}H\ket{x}$, corresponds exactly to the cost of the corresponding variable assignment $C(x)$.

QAOA is a hybrid classical-quantum optimization process. The outputs of a certain *parameterized* quantum circuit are fed to a classical optimizer, which then suggests a new set of parameters. This process is repeated until the classical optimizer terminates. A final set of samples is taken from the circuit with the optimized parameters, and the sample with the best cost is taken as the solution to the optimization problem.

The circuit is constructed from a layer of Hadamard gates followed by alternating *cost* and *mixer* layers. The cost layer is $U_{C}(\gamma ) = \exp (-i\gamma H)$. There are many choices for a suitable mixer layer; we use $U_{M}(\beta ) = \exp (-i\beta \sum _{i} X_{i})$. The number of such layers is a hyperparameter $p$. In the limit as $p \rightarrow \infty$, QAOA approaches adiabatic quantum computing, which is known to be universal for quantum computing [26]. In contrast, for $p=1$, QAOA is known to be classically simulable under certain circumstances [27]. In the absence of noise, increasing $p$ should increase the convergence rate of QAOA. In practice, the effects of noise mean that a moderate $p$ value is most likely to be effective.

### Classical simulation of quantum adiabatic optimization algorithm

We perform a thorough classical simulation of the QAOA algorithm for tangle resolution. We primarily use the *Qiskit* library, including the GPU-enabled *Aer* simulation package. Simulations are run using up to 4 Nvidia H-100 GPU units and up to 512 GiB of RAM. The *Qiskit* library enables heterogeneous GPU/CPU simulations at large scales, enabling full statevector simulations up to 35 qubits on our hardware.

All of our simulations contain shot noise, i.e. at each iteration, a finite number of samples are taken from the probability distribution according to the final statevector. The classical optimizer is then given only these samples from which to estimate the progress of the optimization.

We find that the convergence of our experiments is greatly improved by using conditional value at risk (CVaR) as the accumulation function, instead of a more traditional energy minimization. In this setting, only the top-performing samples are taken into consideration when evaluating the merit of the QAOA parameters. CVaR only cares about “high value” samples with low energy, and high energy outliers are discarded. Recent studies have justified CVaR both analytically [28] and empirically [28–30].

Key Points

- Using pangenomes to resolve short-read data avoids linear reference bias, i.e. unavoidable in traditional reference-guided mapping.
- Compared with *de novo* assembly, pangenome-guided assemblies report far fewer contigs and cover a greater amount of the true genome, with only a small increase in the number of false joins.
- Our novel optimization-based approach achieves comparable results to bleeding-edge exhaustive methods, and will remain feasible as problem size increases.
- Our methods are designed to run efficiently on quantum computers as quantum technology improves; already, quantum annealing methods achieve good results on moderate-sized instances.

## Supplementary Material

bbag084_Pangenome_guided_sequence_assembly_via_binary_optimisation_supp_v4

## Data Availability

All code used to produce data and plots is available on GitHub at https://github.com/jkbonfield/qpg.

## Notes

Author Note: all authors are part of a research collaboration participating in the Wellcome Leap Q4Bio programme, of which Sergii Strelchuk is the main PI and Richard Durbin a co-PI.
