# Supplementary material for: Pangenome-guided sequence assembly via binary optimization
Source: Brief Bioinform. 2026 Feb 26;27(1):bbag084. doi: 10.1093/bib/bbag084 (PMC12936794; doi:10.1093/bib/bbag084)
Supplement: bbag084_Pangenome_guided_sequence_assembly_via_binary_optimisation_supp_v4 [file bbag084_pangenome_guided_sequence_assembly_via_binary_optimisation_supp_v4.pdf]

Supplementary Information

S1 A primer on quantum computing and quantum optimisation

Quantum computing (QC) is an emergent technology that uses the principles of quantum mechanics to perform certain tasks faster than is possible on traditional, classical computers. For example, quantum computers can find the prime factors of large numbers exponentially faster than the best classical algorithms known to computer science. Key physical properties that QC exploits are *superposition* and *entanglement*. Superposition refers to the ability of quantum bits, or *qubits*, are able to exist in both the "0" and "1" state *at the same time*. Intuitively (and extremely informally!), this opens the door for quantum speed-ups by allowing functions to be computed on many inputs simultaneously. Entanglement is a phenomenon in which the state of each particle in some collection cannot be described independently of the others. Acting on one particle in the collection then leads to the "spooky action at a distance" described by Einstein.

A quantum process is most commonly described in the quantum circuit model. In analogy with classical computing circuits, a certain input state is acted on by a sequence of quantum logic gates, which are usually restricted to act on at most 2 qubits. At the end, readout is achieved by taking a measurement of some or all of the qubits. Quantum measurements are probabilistic, outputting a "0" or "1" with probabilities in accordance with the state before the measurement was taken. The output of a quantum circuit is therefore simply a bit string, identical to those produced by classical circuits. The end goal of quantum algorithms is to move as much probability mass as possible onto bit strings that encode the correct answer for a given input.

While the potential for quantum advantages is appealing, there are downsides to the use of QC, at least currently. Quantum devices are inherently noisy and error-prone. The downside of exploiting superposition is that quantum states are analogue signals, rather than the digital state of classical bits. Interference from the environment on the physical system that contains the quantum computer causes the quantum states to drift. Algorithms often rely on constructive or destructive interference to shift probability onto desired states, and this drift reduces how effectively that process works. Also, quantum logic gates are prone to errors, with 2-qubit gates failing at a rate of around 4×10^{-4} on leading hardware. While certain error mitigation strategies are possible, and quantum error correction is promising in the long run, errors are unavoidable on current hardware. Moreover, quantum computers are limited in size, with no more than 156 qubits present on commercially available hardware. The presence of noise and the lack of qubits mean that quantum algorithms for current hardware must be designed to be resource-efficient. No more than a few thousand gates can be run without the combination of errors and noise destroying any information.

There are several promising avenues for algorithms that might experimentally demonstrate the utility of quantum computers on current or near-term hardware. Arguably chief among them is quantum optimisation and the poster-child algorithm, the Quantum Approximate Optimisation Algorithm (QAOA). QAOA is a hybrid classical-quantum algorithm, consisting of a quantum circuit whose gates are parametrised by a set of variables, and a classical optimiser. The quantum circuit is sampled several times, and these samples are used to estimate a cost function. The cost function is then used by the classical optimiser to suggest new parameters for the quantum circuit. The process is iterated until the optimiser reports convergence or some other termination criterion, such as the total number of samples, is reached. QAOA experiments have been successfully run with up to 127 qubits.

Although quantum circuit model hardware is currently limited, there is an intermediate technology that allows for preliminary experimentation. Quantum Annealing (QA) is an optimisation process that can directly be applied to combinatorial optimisation problems. Quantum annealers exist outside of the quantum circuit model and are not able to perform general computational tasks. However, they are well-suited to the problems we consider in this work. Moreover, implementations of QA, most notably by *D-Wave*, are much more advanced than their circuit counterparts. *D-Wave*'s latest system has over 4,400 qubits, allowing classically-challenging problems to be tackled by quantum hardware. While there is some debate as to the validity of these systems as true quantum computers, we view them as a useful indication of the future power

of QC.

S2 Future research directions

It is tempting to draw comparisons and make projections about how indicative *D-Wave*'s success might be for the potential advantages of QAOA methods. However, there is no strong evidence to suggest that successful performance on a *D-Wave* machine provides any meaningful insight into the performance of QAOA on the same QUBO formulation. A thorough study of this connection remains out of reach due to the limited number of qubits and the shallow gate depths available on current circuit-based quantum platforms. Moreover, for this problem, running QAOA with a QUBO Hamiltonian may not be the most efficient approach, as the required number of qubits scales unfavourably with the size of the problem instance. Instead, one should consider methods that bypass the QUBO formulation altogether and encode the objective directly into the Hamiltonian in a fundamentally different way.

One drawback of the current optimisation formulation is the lack of edge information. When we have graphs with multiple paths through the same set of nodes, it can be impossible to distinguish nodes A+ B+ C+ from A+ B- C+. *Pathfinder* combines edge weights with node weights to resolve these conflicts. Experiments using *pathfinder* with and without edge weights have shown that using this information has a minor impact on most statistics, but can reduce the number of false joins by up to a third.

While it is straightforward to include an edge weight term in the abstract optimisation cost functions of eqs. (1) to (3), it is not clear how to translate such terms into a QUBO. The information of whether an edge was traversed at a certain time is inherently quadratic, given by terms $x_{t,i}x_{t+1,j}$. To obtain a cost function that is minimised when the number of traversals of an edge is close to the weight of that edge requires terms like $\sum_t \left(x_{t,i}x_{t+1,j} - w(i,j)\right)^2$ for each $(i,j) \in E$. These higher-order terms incur overheads in the number of variables if they are re-expressed as quadratic terms. A local graph preprocessing step to remove zero-weight edges that do not break graph connectivity is likely to achieve an approximate equivalent without the need to incorporate edge data directly into the QUBO model.

All graph path finding methods are impacted by the quality of the input kmer counts. Further improvements can be made to these tools, particularly concerning non-unique mapping, which would improve the quality of all solvers. However, there is further missing information yet to be considered, where larger sequence fragments trace a path through many small nodes, forming longer-range constraints rather than the nearest neighbour ones encoded in the graph edge counts.

The kmer mapping software used to generate node weights does not use Illumina read-pairing metrics. In a traditional linear reference alignment, the read pairing is an important factor for resolving some repeats, and the same could also be applied to pangenome mapping. Pairing information could potentially also be used in inferring paths, utilising knowledge of the template size distribution.

A major drawback of the QUBO formulation is the $\mathcal{O}(NT) = \mathcal{O}(N^2)$ scaling in the number of variables, a nearly quadratic increase compared to the natural $\mathcal{O}(T\log(N)) = \mathcal{O}(N\log(N))$ number of variables required to encode a length-T walk on N nodes in binary. While such overheads are permissible in a complexity-theoretic sense, practically, they can lead to huge increases in the difficulty of finding optimal solutions. Moreover, the number of qubits required essentially precludes industry-scale problems from being tackled with our current approach on a quantum computer in the near term.

One possible direction that improves resource usage and also allows edge weight data to be included is the use of Higher-order Unconstrained Binary Optimisation (HUBO) variants. HUBO formulations are more expressive than QUBO, with terms containing products of arbitrarily many binary variables; the downside is that (classically) solving them is generally more difficult and less well-explored in the literature. Notably, however, HUBO may not have such a solution overhead when mapped to QAOA algorithms on quantum computers. In that setting, each layer of the cost Hamiltonian now contains multi-qubit Z rotations rather than single- and two-qubit rotations only. These gates need to be compiled down to 2-qubit gates, but constructions that are linear in the number of qubits involved exist [1]. In this setting, the inclusion of

edge weight data requires the implementation of more multi-qubit interactions, which in turn increases the circuit depth. Notably, there are no overheads in the number of qubits, which is a major limiting factor in current-term quantum hardware. We could also consider Prog-QAOA [2] formulations, which directly encode optimisation problems into QAOA cost Hamiltonians, without going through a QUBO or HUBO step. We expect that either of these 2 encodings could achieve $\mathcal{O}(N \log(N))$ qubit scaling, at the cost of increased circuit depth.

Another potential improvement is to modify the cost function to place greater emphasis on larger nodes. Larger nodes have less variance in the number of unique kmer hits, so we should have more confidence that the annotated values represent the true copy number. Also, they represent a larger part of the underlying sequence, so they are more important to get correct. For example, we might modify the objective of Tangle Resolution, given in eq. (1), to

$$C_G(W) = \sum_{v \in V} \log(L(v)) \Big(\#W(v) - w(v) \Big)^2, \tag{1}$$

where $L(v)$ is the length of the sequence stored in node v.

We will also investigate the performance of the QAOA solvers using hardware experiments rather than simulations. On hardware with up to 156 qubits and an encoding that uses only $\mathcal{O}(N \log(N))$, we could encode problems with around 30 nodes. These are large enough to be classically non-trivial for certain hard instances, and would provide strong evidence for quantum utility in this domain if the quantum algorithms performed well.

Finally, we will properly test and benchmark the performance of the Diploid Tangle Resolution solvers. In this setting, it is more subtle to evaluate the quality of the solution, as the two paths might erroneously swap which allele they are representing. We will look to incorporate methodology from existing phasing software to aid in solution refinement and evaluation.

S3 Details of node weight assignment methods

A critical component of the robustness of path finding is the quality of the input node coverage scores. These are processed by the solvers to yield a node copy-number, but the GFAv1 specification permits a kmer count in the `SC:i` tag. Depth can be converted to coverage by dividing by the length (or expected observable length), and depth to coverage by observation of average depth across nodes or knowledge of the input sequencing depth.

S3.1 Minigraph

The *minigraph* [3] option `--cov` may be used to redisplay the input GFA file with a report of the percentage of kmers observed in the `dc:f` tag. This is modified to the standard kmer-count `KC:i` tag by multiplying by the node length. For edges, `dc:f` is converted to the `EC:i` tag.

This also permits visualisation within the *Bandage* tool and for normalisation with other tool chains.

The command line options for synthetic short read data are:

```
minigraph -x sr -j 0.01 --cov $gfa reads.fa > reads.mg
perl -we script-to-add-tags reads.mg > reads.gfa
```

S3.2 Kmer2node

The basic strategy of *kmer2node* is to build a kmer index of the sequence in each node and then to match every kmer in each short read against this computed index.

Input genomes to build the graph, with spacing for visualisation purposes only:

```
CAT ACTCC CCC GGACAAGG TAG
CAT ACACC CCC GGTCATGG TAG
CAT GGTGG CCC  ACATT   TAG
CAT GGAGG CCC  ACTTT   TAG
```

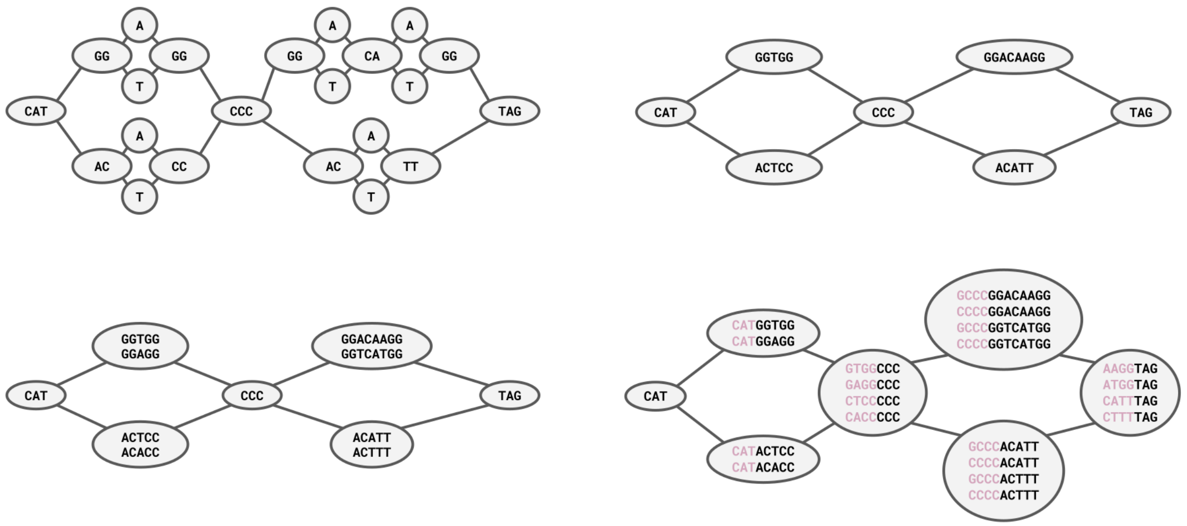

Figure S1: A pictorial view of *kmer2node* processing on a graph built from 4 short example genomes. On the top left we have a fully complete pangenome capturing variation at the individual base level (*minigraph-cactus* style). Top right is a lossy graph with small variants collapsed into representative nodes (*minigraph* style). Bottom left is produced by aligning the full length genomes used to build the graph back to the graph and tracking which sequence substrings align to each node. Bottom right is after recursively walking back through the graph from each node to generate $k-1$ prefix bases.

The pangenome graphs we process come from *minigraph*, which does not produce new nodes for minor sequence variation, leading to imprecise mapping. This is beneficial as it reduces the complexity of the graphs to resolve, making the problem more amenable to the quantum environment, but it also means kmer matching may miss some hits. We do, however, have the original input genomes that were used to build the pangenome. By aligning these back to the pangenome graph with *GraphAligner*, we can get a path of node names along with a CIGAR string. Combined, these yield the exact sequence from the training genomes that covers each node rather than the single one reported by *minigraph*. This ancillary "nodeseq" file is also indexed, improving the mapping accuracy of *kmer2node*. See fig. S1 for a pictorial demonstration of these steps.

The repetitive nature of the sequences in the graph nodes means that not all kmers can be uniquely placed. A unique node sequence of length L will have $L-k+1$ kmers of length k. However, during indexing, we track which kmers are unique and which are not to record an expected number of unique kmers per node sequence. This is used during mapping to compensate for non-unique segments. Additionally, a sequence of kmers from a short-read that spans unique to non-unique and back to unique within the same graph node means we can still identify the internal non-unique kmers as belonging to that node. We can additionally rescue some non-unique kmers at the start and ends of nodes by a similar strategy. Finally, short reads that transition from one node to another node with non-unique kmers may still be disambiguated if the graph edges indicate only one combination of nodes is possible.

At present, *kmer2node* does not exploit knowledge of paired Illumina reads, but, in principle, these can also be used to resolve some repeat locations. Improved handling of repetitive kmers should also permit mapping qualities to be produced, providing partially qualified evidence for more paths. For now, *kmer2node* restricts itself to being conservative in reporting matches.

For this work, the command lines used are:

```
GraphAligner -g $gfa -f train.fa -x vg -a pop.gaf
for k in 75 35 20;do
    gaf2nodeseq2.pl pop.gaf train.fa $gfa $k > $gfa.nodeseq.$k
    kmer2node4 -G $gfa -U -k$k $gfa.nodeseq.$k reads.fa -E /dev/stdout > reads.$k
done
merge_kmer2node.pl $gfa reads.k* | tag_gfa_km.pl $gfa > reads.gfa
```

The tool was written purely for research into this problem and is not yet considered production quality. It also maps every kmer and is considerably slower than comparable minimiser techniques. It can be found in the qpg tangle GitHub repository along with the evaluation code.

S3.3 GraphAligner

GraphAligner [4] cannot emit kmer count data. However, the standard alignment format does include an alignment path indicating the nodes used, along with a CIGAR string indicating which strings of bases map against which node. Nodes internal to a multi-node path string (e.g. "s2" and "s3" in ">s1 >s2 >s3 >s4") will have their kmer count incremented by the length of the node. Nodes on the end of the path string have their kmer count incremented according to the relative proportion of the remaining sequence. This is used to fill out `KC` and `SC` auxiliary tags on GFA "S" nodes and `EC` on "L" edges.

Note *GraphAligner* cannot distinguish between primary and secondary alignments. A sequence may align to multiple locations without regard to which is best. The *tag_gfa_ga.pl* program attempts to identify the best single alignment, but this may still be a non-uniquely mapped repeat region. This gives rise to higher average node coverage than *minigraph* and *kmer2node*, but with poorer specificity. This appears to help *pathfinder*, which does not like many zero-depth nodes, but hampers the QUBO formulations.

The parameters used for running GraphAligner are:

```
GraphAligner --min-alignment-score 90 --seeds-minimizer-ignore-frequent 1e-2 \
    -f $gfa -f reads.fa -x vg -a reads.gaf
tag_gfa_ga.pl $gfa 10 reads.gaf > reads.gfa
```

S3.4 Giraffe

Unlike the other tools used vg's *giraffe* [5] aligner is a dedicated short-read aligner, capable of understanding Illumina read pairs. However, our synthetic data is not currently simulated read-pairs.

Giraffe does not emit kmer data, but it can be used to produce the same GAF format as *GraphAligner*. We then use the same pipeline to process the GAF node strings into fake kmer data. One key difference, however, is *giraffe* creates robust mapping qualities, making it easier to select the best aligned copy when multiple alignments are reported, and to filter out poor matches.

The command lines for running *vg giraffe* are:

```
vg autoindex --workflow giraffe --prefix $gfa -g $gfa
vg giraffe -Z $gfa.giraffe.gbz --named-coordinates -f read.fa -o gaf > read.gaf
tag_gfa_ga.pl $gfa 10 reads.gaf > reads.gfa
```

S4 Preliminary results of graph edge preprocessing

The annotation methods can provide coverage data for both nodes and edges, with edge data being produced when we observe a single sequence of kmers transitioning from one node to another. A significant source of sequence "breaks" arises from inversions where a sequence has been observed in the pangenome to be flipped

in orientation. meaning we have a graph where a node can be traversed in either orientation. Only edge information can be used to decide which orientation should be utilised. However, the QUBO formulations do not currently take into account edge weights, which puts them at a disadvantage compared to *pathfinder*. While a formal inclusion of edge weights is possible, it adds complexity to the model.

We implemented a local graph preprocessing step that observes cases where a node has multiple input and output edges and at least one input and one output has non-zero edge weight and non-zero node weight. In this case, any additional edges with zero weights can be deemed to be superfluous and removing them will not break graph connectivity. The results can be seen in table 1, which demonstrates a significant drop in the number of breaks and a slight increase in percentage covered and used.

Annotator	Trim	%Covered	%Used	No.Contigs	No.Breaks	No.Indel	No.Diff	%Identity
kmer2node	N	86.5	91.6	2.6	2.4	0.5	0.3	98.7
kmer2node	Y	87.0	92.6	2.6	2.1	0.6	0.3	98.7
minigraph	N	84.2	91.7	4.4	2.5	0.4	0.2	99.2
minigraph	Y	84.1	92.1	4.3	2.0	0.4	0.1	99.2
GraphAligner	N	87.2	77.2	1.6	5.9	0.9	0.3	98.4
GraphAligner	Y	89.4	78.0	1.6	5.4	0.9	0.3	98.5
giraffe	N	87.6	76.2	1.6	6.7	1.0	0.2	98.6
giraffe	Y	88.5	78.5	1.6	5.8	1.0	0.3	98.5

Table 1: Results of the MQLib solver running for 30 seconds on 51 graphs aligning 5 sequences each, with and without edge trimming enabled. Results are with the consensus optimisation applied.

S5 Preliminary results of hybrid assembly and kmer mapping

Some synthetic problem examples demonstrate *de novo* assembly as the most accurate genome reconstruction, while others suggest that k-mer mapping techniques are the best. We can run *syncasm* to produce an assembly graph with sequence in the nodes. Each node is a conservatively assembled contiguous sequence (a contig), with the edges holding possible alternatives caused by either multiple haplotypes or uncertainty arising from repeated sequences. Using the assembled contigs as input to the pangenome mapping (normalised for our expected depth), optionally alongside the raw sequence fragments, can potentially give us the best of both prior solutions.

Preliminary evidence in table 2 demonstrates a modest improvement in accuracy, particularly when combined with *kmer2node*.

References

[1] Adam Glos, Aleksandra Krawiec, and Zoltán Zimborás. *Space-efficient binary optimization for variational computing*. en. arXiv:2009.07309 [cond-mat, physics:quant-ph]. Sept. 2020. URL: http://arxiv.org/abs/2009.07309 (visited on 05/31/2024).

[2] Bence Bakó, Adam Glos, Özlem Salehi, et al. "Prog-QAOA: Framework for resource-efficient quantum optimization through classical programs". In: *Quantum* 9 (Mar. 2025). arXiv:2209.03386 [quant-ph], p. 1663. ISSN: 2521-327X. DOI: 10.22331/q-2025-03-20-1663. URL: http://arxiv.org/abs/2209.03386 (visited on 07/22/2025).

[3] Heng Li, Xiaowen Feng, and Chong Chu. "The design and construction of reference pangenome graphs with minigraph". In: *Genome Biology* 21.1 (Oct. 2020), p. 265. ISSN: 1474-760X. DOI: 10.1186/s13059-020-02168-z. URL: https://doi.org/10.1186/s13059-020-02168-z.

Annotator	Input	%Covered	%Used	No.Contigs	No.Breaks	No.Indel	No.Diff	%Identity
kmer2node	R	88.4	94.0	2.6	1.5	0.6	0.3	98.7
kmer2node	C	85.3	93.7	3.3	1.6	0.4	0.2	99.0
kmer2node	R+C	89.4	94.0	2.3	1.5	0.6	0.3	98.6
GraphAligner	R	93.1	92.7	1.6	1.6	0.8	0.3	98.6
GraphAligner	C	90.8	93.8	2.2	1.5	0.6	0.2	98.9
GraphAligner	R+C	93.2	92.6	1.6	1.7	0.8	0.3	98.6
syncasm	C	91.6	86.2	31.3	0.0	0.1	0.0	100.0

Table 2: Demonstration of the impact of combining *de novo* assembly and kmer mapping techniques. The input column lists "R" for raw reads and "C" for contigs produced from running *syncasm* on the raw reads. For *kmer2node* we show that raw data and contig data alone are poorer than the combination of the two, while with *GraphAligner* there is minimal change. We also include an assessment of *syncasm* directly without using the pangenome. *Kmer2node* figures are from the optimised consensus.

[4] Mikko Rautiainen and Tobias Marschall. "GraphAligner: rapid and versatile sequence-to-graph alignment". In: *Genome Biology* 21.1 (Sept. 2020), p. 253. ISSN: 1474-760X. DOI: `10.1186/s13059-020-02157-2`. URL: `https://doi.org/10.1186/s13059-020-02157-2`.

[5] Jouni Sirén, Jean Monlong, Xian Chang, et al. "Pangenomics enables genotyping of known structural variants in 5202 diverse genomes". In: *Science* 374.6574 (2021). Publisher: American Association for the Advancement of Science, abg8871. DOI: `10.1126/science.abg8871`. URL: `https://doi.org/10.1126/science.abg8871` (visited on 07/22/2025).
